# A Case of Acute Massive Bioprosthetic Mitral Valve Thrombosis Leading to Fulminant Heart Failure

**DOI:** 10.1155/2020/7842591

**Published:** 2020-01-11

**Authors:** Zeid Nesheiwat, Pinang Shastri, Rohit Vyas, Cameron Burmeister, Robert Grande, Hazem Malas

**Affiliations:** ^1^Department of Internal Medicine, The University of Toledo College of Medicine, 3000 Arlington Avenue, Toledo, Ohio 43614, USA; ^2^Division of Cardiovascular Medicine, Toledo Hospital, 2142 N Cove Boulevard, Toledo, Ohio 43606, USA

## Abstract

Bioprosthetic valve thrombosis has been considered to be extremely unlikely, typically freeing patients from the potential complications of long-term anticoagulation. However, there have been several documented cases of bioprosthetic valve thrombosis and there are concerns that its incidence may be underreported. Experience with diagnosis and management of this condition is limited. Here, we present a case of acute massive bioprosthetic mitral thrombosis manifesting as fulminant heart failure.

## 1. Introduction

Over 150,000 valve replacements are performed annually in the United States and over 80% of these procedures utilize bioprosthetic valves [1]. The large shift from mechanical valves to BPVs is related to the growing high-risk population that require valve replacement, the appeal of short-term anticoagulation therapy, and options for minimally invasive procedures making them a good option for patients who are poor surgical candidates or have an increased risk of bleeding. BPV replacements are relatively safe procedures with a minimal rate of serious complications, typically 2-3%, which include thromboembolic events, structural valve dysfunction, endocarditis, and hemolysis [2]. Current guidelines support short-term oral anticoagulation with or without aspirin as well as dual antiplatelet therapy following transcatheter aortic valve replacement [1].

Valve thrombosis is a rare and life-threatening complication of valve replacement surgery. While more common in mechanical valves, valve thrombosis can occur in bioprosthetic valves with severe consequences. The diagnosis remains challenging mainly due to a general lack of awareness of its existence [2]. Typically, bioprosthetic valve thrombosis occurs within 1-2 years of implantation with some studies showing as early at 6 months postimplantation [1, 3]. Treatment options include surgical excision and reimplantation, fibrinolysis, and anticoagulation. Here, we present a case of acute bioprosthetic mitral valve thrombosis with fulminant heart failure requiring emergent invasive cardiac surgery.

## 2. Case Presentation

**Figure 1:**
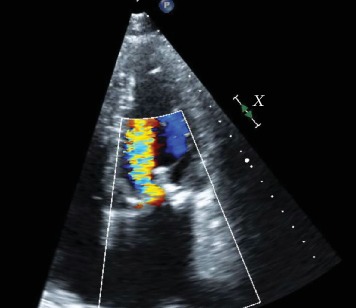
Severe mitral regurgitation noted on four-chamber apical view.

**Figure 2:**
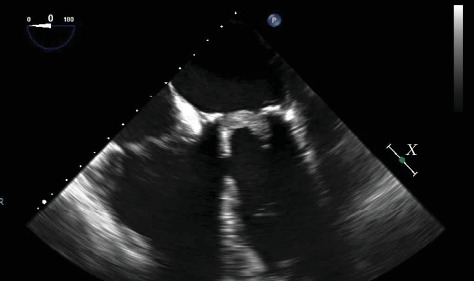
Four-chamber apical view of bioprosthetic mitral valve thrombosis.

**Figure 3:**
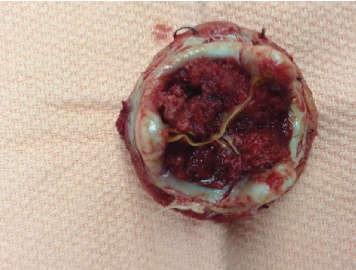
Massive thrombus completely obstructing the bioprosthetic valve as seen in the postoperative photo.

A 65-year-old male with a past medical history significant for hypertension, asthma, and minimally invasive bioprosthetic mitral valve replacement with a 33 mm mosaic mitral porcine valve one year prior presented with severe respiratory distress requiring emergent intubation. Initial electrocardiogram (EKG) revealed sinus rhythm with left anterior fascicular block, right bundle branch block, and evidence of left atrial enlargement. Initial troponin was normal at 0.04 ng/mL, but CK-MB and total CK were elevated to 21.2 ng/mL and 308 U/L, respectively. Anticoagulation studies revealed an INR of 1.0, a PTT of 28, and a PT of 10.9. Following transfer to the intensive care unit, the patient was started immediately on heparin infusion for suspicion of acute coronary syndrome (ACS); computed tomography angiogram (CTA) of the chest revealed moderate patchy ground-glass and nodular airspace opacities with interstitial edema and cardiomegaly. Transesophageal echocardiogram (TEE) was performed showing an acute thrombosis of the bioprosthetic mitral valve with severe mitral stenosis and regurgitation exhibiting a mean gradient of 20.1 mmHg and valve area of 0.68 cm^2^ (Figure 1). Of note, the patient had already completed 6 months of anticoagulation following his bioprosthetic mitral valve replacement and was no longer on anticoagulation. He was also found to have near complete leaflet immobility and subsequently underwent emergent redo mitral valve surgery utilizing a 33 mm St. Jude mechanical prosthesis. The valve was noted to have a massive amount of material on the ventricular side of each cusp (Figures 2 and 3) with pathology findings consistent with thrombosis. Following valve replacement, intraoperative TEE showed that the mitral valve mean gradient had improved to 4 mmHg. The procedure was well-tolerated, and the patient was successfully extubated and transitioned to warfarin for anticoagulation. Hypercoagulable workup was negative, and he was doing well on follow-up.

## 3. Discussion

Despite the rising popularity and efficacy of bioprosthetic valve implantation, the incidence rate of acute complications is often underreported and many patients who undergo acute intervention lack proper echocardiographic follow-up. A 2015 Mayo Clinic study identified bioprosthetic mitral valve thrombosis in 11% of explanted bioprosthetic mitral valves; in addition, this study revealed that 65% of all reoperations for bioprosthetic mitral valves occurred more than one year after implantation and up to 15% of these reoperations occurred more than five years after the initial implantation [4]. This study estimated the incidence of bioprosthetic mitral valve thrombosis at one percent, but this was based solely on a subset of the cohort of patients who had follow-up echocardiography. Almost one-half of the patients did not have proper echocardiographic follow-up [4].

Independent predictors of bioprosthetic mitral valve thrombosis include a combination of clinical and echocardiographic findings such as increased echo-doppler gradient from baseline, underlying coagulopathy, left ventricular dysfunction, prosthetic mismatch, paroxysmal atrial fibrillation, subtherapeutic International Normalized Ratio (INR), increased cusp thickness, previous Maze procedure, and abnormal cusp mobility [2, 5]. A paper by Egbe et al. recently proposed a model for diagnosing bioprosthetic valve thrombosis by combining thee echocardiographic predictors: a 50% increase in transvalvular gradient compared to baseline, increase cusp thickness (>2 mm), and abnormal cusp mobility. The study concluded that applying this model yielded a 72% sensitivity and 90% specificity [2]. Further studies addressing incidence and complication rates in patients with bioprosthetic mitral valve thrombosis will allow for the elucidation of proper follow-up intervals and treatment strategies including anticoagulation and screening intervals. Typically, the highest risk for bioprosthetic mitral valve thrombosis is within the first ten days of implantation. Our patient presented with massive bioprosthetic mitral valve thrombosis one year after transcatheter replacement with no echocardiographic follow-up following valve placement signifying that more frequent assessments in the first several years after replacement may be prudent.

It would also be beneficial to compare the effect of different antithrombotic regiments on long-term clinical outcomes after transcatheter valve replacement. Previously reported cases of bioprosthetic mitral valve thrombosis have resolved with anticoagulation therapy suggesting clot burden as a significant factor in determining optimal treatment regimens [6]. While anticoagulation is typically prescribed in the short-term setting, typically the first few months, to prevent acute complications such as thromboembolic events, cases of late valve thrombosis sometimes up to two years after valve implantation, including the above, suggest that severe complications are still possible with bioprosthetic mitral valves leading to potentially fatal outcomes [5]. The significance of establishing guidelines regarding proper screening and medical therapy is especially relevant due to recent studies supporting transcatheter valve replacement (TVR) in low-risk patients [7]. A recent 2019 meta-analysis assessing outcomes between the transcatheter versus the surgical approach of valve replacements in low-risk patients found that the transcatheter approach was associated with a significantly lower risk of all-cause death and cardiovascular death after one year [8]. The transcatheter approach for valve replacement has been of growing popularity and is slowly becoming the standard of care. However, further studies regarding optimal duration and choice of anticoagulation will be necessary in order to establish more stringent guidelines for their therapy.
